# Green, Yellow, and Red Fluorescent Proteins as Markers for Bacterial Isolates from Mosquito Midguts

**DOI:** 10.3390/insects10020049

**Published:** 2019-02-03

**Authors:** Ephantus J. Muturi, Jose L. Ramirez, Chang-Hyun Kim

**Affiliations:** 1 Crop Bioprotection Research Unit, U.S. Department of Agriculture, Agricultural Research Service, National Center for Agricultural Utilization Research, 1815 N. University, St. Peoria, IL 61604, USA; Jose.Ramirez@ars.usda.gov; 2 Illinois Natural History Survey, Prairie Research Institute, University of Illinois at Urbana-Champaign, Champaign, IL 61820, USA; maraychk@illinois.edu

**Keywords:** *Ochlerotatus triseriatus*, gut bacteria, fluorescent proteins

## Abstract

The growing awareness that microbial symbionts residing in mosquito midguts can interrupt transmission of vector-borne diseases has stimulated interest in understanding their potential role in mosquito biology. Fluorescent proteins are powerful molecular markers that provide for detailed analysis of the function and behavior of specific midgut bacterial isolates without disturbing the normal gut microbiota. The aim of this study was to label bacterial isolates from the midgut of *Ochlerotatus triseriatus*, the primary vector of La Crosse virus, with green, yellow, and red fluorescent proteins (GFP, YFP, RFP) via electroporation. We also assessed the stability of GFP-, YFP-, and RFP-bearing plasmids and their effect on bacterial growth. Seven of eleven bacterial species could not be labeled despite several attempts. Labeling of *Escherichia coli* and *Enterobacter cloacae* was successfully achieved with all three fluorescent proteins. In contrast, labeling of *Aerococcus viridans* was achieved with GFP only and labeling of *Aeromonas hydrophila* was achieved with GFP and YFP only. The stability of GFP plasmid varied among bacterial species with *A. hydrophila* followed by *E. cloacae* having the most stable GFP label. In contrast, YFP and RFP plasmids were very stable in all bacterial species possessing these labels. GFP plasmid reduced the growth of labeled strains relative to wild type but this effect was not evident in YFP and RFP plasmids. These findings suggest that some mosquito midgut bacterial isolates can effectively be labeled with GFP, YFP and RFP plasmids allowing non-destructive studies on their functions within the vector.

## 1. Introduction

Mosquito-borne diseases such as malaria, dengue, and West Nile virus contribute a significant portion of the estimated global burden of all infectious diseases. Over the last few decades, the global incidence and geographic range of these diseases have expanded dramatically, primarily due to globalization and climate change [1]. Suppression of vector populations with insecticides is a proven and effective strategy for prevention and management of mosquito-borne diseases, but the urgent need for alternative approaches has become apparent due to the emergence of insecticide resistance and concerns regarding the negative environmental and public health consequences of insecticide use.

The microbial communities in mosquito midgut have attracted significant research interest as novel tools for combating mosquito-borne diseases under a multifaceted approach referred to as “symbiotic control” [2]. This research is motivated by the findings that certain midgut bacterial species can enhance or suppress viral and parasitic infections in mosquitoes [3,4,5,6,7,8] and that midgut bacterial symbionts could be genetically-modified to express anti-pathogen molecules that reduce vector competence (paratransgenesis) [9,10,11]. However, while significant progress has been made in the characterization of microbial communities that reside in mosquito midguts [5,12,13,14], the application of these microbes in disease control is hampered by our limited understanding of their functional roles in mosquito biology.

A major challenge in studying the function of specific midgut bacterial isolates on mosquito biology is tracking and distinguishing inoculated bacteria from indigenous microbial population. Traditionally, this challenge has been overcome by clearing the normal gut flora with antibiotic therapy administered orally and then introducing the target bacteria via blood meal or sugar meal and examining the outcome [3,6,15,16]. This approach has yielded important insights into mosquito–microbe interactions and their potential impact on pathogen transmission. However, antibiotic therapy may have deleterious effects on insect tissues and overall fitness, and partitioning these effects from specific effects of introduced microbes can be challenging [17,18]. Furthermore, the role of the target bacterial species is typically examined in the absence of other bacterial species, yet the mosquito midgut is a microbiologically complex ecosystem. Thus, there is an urgent need for simple, rapid, precise, and sensitive methods for studying mosquito–microbe interactions under realistic environmental conditions.

Labeling microbes with green fluorescent protein (GFP) is a popular method for tracking living bacterial cells in situ [19,20]. GFP was discovered from the jellyfish *Aequorea victoria* in the 1960s, and its gene has since been isolated, cloned, and expressed in both eukaryotic and prokaryotic hosts [21,22]. A GFP-marker allows the study of the function and behavior (persistence, survival, colonization) of specific bacterial isolates in their natural habitats (e.g., mosquito midgut) nondestructively and without the addition of exogenous substrates. GFP-marked bacterial cells can be visualized by using standard microscopes equipped with commonly available fluorescent filter sets [21]. Because only one copy of GFP gene is present in a single bacterial cell, the abundance of the target bacterial species can be measured by fluorescent intensity measurements, fluorescent correlation spectroscopy, or quantitative polymerase chain reaction (qPCR). Recent advances in fluorescent protein engineering have also produced the red fluorescent protein (RFP) and the blue, cyan, and yellow variants of GFP [23]. These spectrally separated fluorescent proteins make it possible to introduce and track the behavior and function of multiple gut bacterial species in the vector.

The objective of this study was to label bacterial species isolated from the midguts of larval and adult populations of *Ochlerotatus triseriatus*, the primary vector of La Crosse encephalitis virus with GFP, yellow fluorescent protein (YFP) and RFP for our mosquito–microbe interaction research projects. We also assessed the stability of GFP, YFP, and RFP plasmid in the labeled bacteria and its effect on bacterial growth. The findings of this study provide a simple and powerful method for studying the fate and functions of specific bacterial isolates in mosquito midguts.

## 2. Materials and Methods

### 2.1. Bacterial Species

A total of 11 bacterial species isolated from the midguts of larval and adult populations of *Oc. triseriatus* collected in Urbana, IL were utilized for transformation. These included *Aerococcus viridans*, *Aeromonas hydrophila*, *Bacillus cereus*, *B. indicus*, *B. firmus*, *B. megaterium*, *Enterobacter cloacae*, *Escherichia coli*, *Exiguobacterium aestuarii*, *Lysinibacillus sphaericus*, and *Pseudomonas fluorescens*. These were the only bacterial species successfully isolated and cultured from *Oc. triseriatus* and hence their selection for this study. Homogenates of pooled midguts of larval or adult *Oc. triseriatus* were cultured on MacConkey agar, Columbia blood agar with 5% sheep blood or brain heart infusion broth. Definitive identification of morphologically distinct bacterial colonies was achieved through PCR amplification and sequencing of a segment of 16S rRNA gene followed by BLAST search. The universal bacteria primers used for this study were Eub338 5-ACT CCT ACG GGA GGC AGC AG-3 and Eub518 5-ATT ACC GCG GCT GCT GG-3. These bacterial species have been maintained by sub-culturing them on fresh Luria-Bertani (LB) agar plate at seven-day interval.

### 2.2. Transformation of Bacterial Species

Transformation by electroporation was carried out using the methods described by Peloquin et al. [24] with slight modifications. Briefly, a colony of one of 11 bacterial species was cultured in 10 mL Luria-Bertani (LB) broth containing 10% Compbooster^®^ (Tritech Research, Los Angeles, CA, USA) at 37 °C with constant shaking at 250 rpm for 20 h. The bacteria culture was washed 4 times with sterile 15% Glycerol solution and centrifugation at 10,000× g for 10 min. The resulting bacterial cell pellet was resuspended in 1 mL of sterile 15% Glycerol solution. Fifty µL aliquot of bacteria suspension was mixed with 2 ng plasmid encoding GFP (Altogen Biosystems, Las Vegas, NV, USA), YFP or RFP (Addgene, Cambridge, MA, USA). The bacteria-plasmid mixture was electroporated using BactoZapper^®^ Cloning Gun^®^ (Tritech Research, Los Angeles, CA, USA). Immediately after electroporation, the bacteria suspension was mixed with 250 µL SOC media and incubated at 37 °C with shaking at 250 rpm for 1 h. The resulting bacteria suspension was spread on a pre-warmed LB agar plate containing kanamycin (100 µg/mL) and incubated overnight at 37 °C and a well-separated bacterial colony was selected.

### 2.3. Plasmid Stability Testing

The stability of GFP, YFP, and RFP-containing plasmids in GFP/YFP/RFP-labeled bacterial strains was confirmed through sequential propagation of labeled bacterial strains in the absence of antibiotic (kanamycin) selection pressure [20,25]. Overnight cultures of GFP/YFP/RFP-labeled strains grown in the presence of kanamycin were used to inoculate Luria-Bertani (LB) broth without kanamycin. Bacteria were grown in kanamycin-free LB broth and were transferred daily (24-h intervals) at a 1:100 dilution into fresh medium for 14 consecutive days. Each day, 10 µL culture samples were spread onto agar plates with kanamycin. The plates were incubated overnight at 37 °C and examined for presence of colonies. Bacterial colonies growing on LB agar plate containing kanamycin were considered to be those possessing the GFP/YFP/RFP plasmids. The GFP plasmid stability was determined by the number of days showing the formation of bacterial colonies on LB agar plate containing kanamycin.

For PCR assays, 1 mL aliquot of overnight culture from each passage was centrifuged at 12,000× g for 10 min. After discarding the supernatant, the remaining bacterial cell pellet was suspended in 100 µL of sterile PBS and bacterial DNA in the suspension was isolated using DNeasy Blood & Tissue Kit (Qiagen, Valencia, CA, USA). The resulting DNA was further diluted 100 fold and 2 µL DNA was utilized for PCR amplifying a segment in each fluorescent protein plasmid flanked by specific primers (Table 1). The primers for RFP and YFP were manually designed based on DNA sequences of RFP (plasmid sequence of pBbE8k-RFP) and YFP (plasmid sequence of pEF3-YFP-2) retrieved from Addgene (Cambridge, MA, USA) website. Amplification of GFP gene segment in GFP-labeled bacterial strains was accomplished using universal primers since available GFP-specific primers were unable to accomplish this (Table 1). The PCR was conducted in a 20-µL reaction mixture containing 10 µL of 2× SensiFAST™ Probe Hi-ROX master mix (Bioline, Taunton, MA, USA), 0.4 µL each of 10 µM forward and reverse primers, 7.2 µL of nuclease free water and 2 µL of template DNA. Thermal cycling conditions on ABI 7300 HT sequence detection system (Applied Biosystems, Foster City, CA, USA) were an initial denaturation step at 95 °C for 5 min, 40 cycles of 95 °C for 60 s, 62 °C for 30 s and 72 °C for 60 s, and a final extension step at 72 °C for 10 min.

The stability of fluorescent protein plasmids was determined by the number of days showing the formation of bacterial colonies on LB agar plate containing kanamycin or the detectable PCR amplicons on gel stained with ethidium bromide.

### 2.4. Growth Curves of Bacteria Transformed with Fluorescent Proteins

In order to generate the growth curves for fluorescently labeled *E. coli*, *E. cloacae*, *A. hydrophila* and *A. viridans*, the bacterial strains were grown overnight in 2 mL BHI broth with kanamycin. Twenty µL of bacterial suspension was inoculated into 1980 µL BHI broth with or without kanamycin and the optical density was measured at 600 nm at 30-min interval for 6.5 to 8 h. Due to slow growth rate and sensitivity of GFP labeled *A. viridans* to kanamycin, the growth curve for this bacterial strain was generated using different reading and incubation time. After growing the bacterial strain in 2 mL BHI broth without kanamycin for two consecutive days, 20 µL bacterial suspension was inoculated into 1980 µL BHI broth without kanamycin and the optical density was measured at 600 nm at 12-h interval for 72 h. Non-transformed wild type strains of each bacterial species were utilized as negative controls for this experiment.

## 3. Results

### 3.1. Construction of Fluorescently Labeled Bacteria

**Figure 1:**
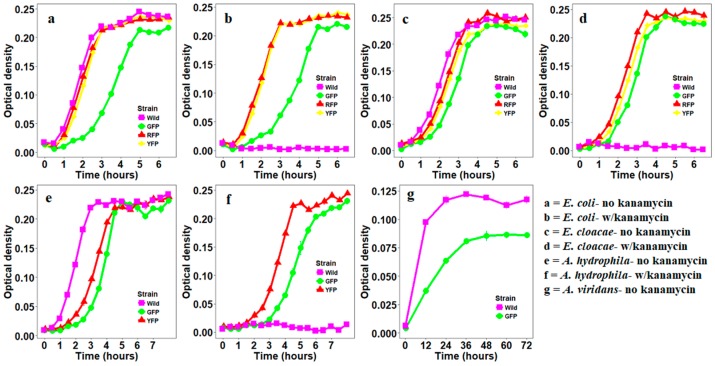
Growth curves for green fluorescent proteins- (GFP-), YPF-, and RFP-labeled bacteria in the presence or absence of antibiotic kanamycin.

Four out of 11 bacterial species were successfully labeled by at least one of the three fluorescent proteins: GFP, YFP, and RFP. These include *E. coli* and *E. cloacae* which were easily labeled by all three fluorescent proteins, *A. hydrophila* which was labeled with GFP and YFP, and *A. viridans* whose labeling was only achieved with GFP. GFP-labeled *E. coli* had a slower growth compared to wild type, YFP- or RFP-labeled *E. coli* (Figure 1a,b). GFP-, YFP-, and RFP-labeled *E. cloacae* had similar growth curves as the wild type with GFP-labeled strain exhibiting the slowest growth rate (Figure 1c,d). GFP- and YFP-labeled *A. hydrophila* and GFP-labeled *A. viridans* had a lower growth rate compared to the wild type (Figure 1e,f). The growth of non-labeled wild type *E.coli*, *E. cloacae* and *A. hydrophila* was inhibited in the presence of kanamycin thus allowing us to efficiently select labeled isolates on selection plates (Figure 1a–f). In contrast, the growth of both wild type and GFP-labeled *A. viridans* was inhibited by kanamycin (Figure 1g).

### 3.2. Stability of GFP Plasmid

**Figure 2:**
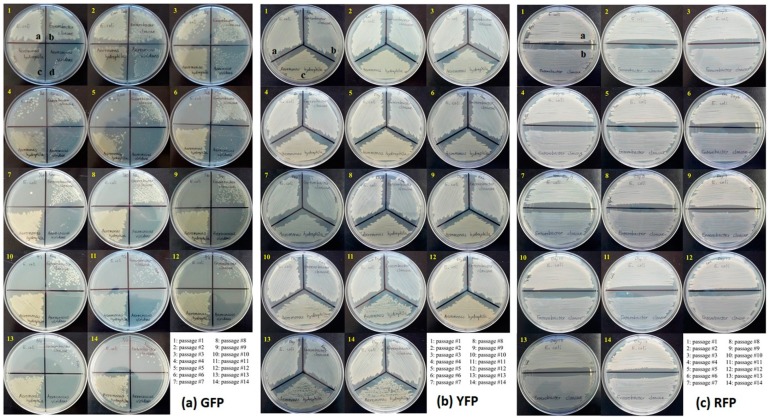
Colony growth characteristics of four GFP-, YFP-, and RFP-labeled bacterial species across 14 passages. a: *Escherichia coli*, b: *Enterobacter cloacae*, c: *Aeromonas hydrophila*, and d: *Aerococcus viridans*.

**Figure 3:**
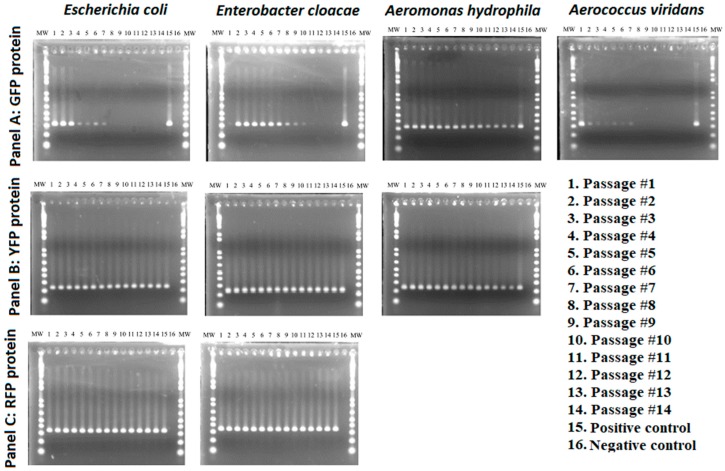
PCR-based detection of GFP-, YFP-, and RFP-labeled bacterial species across 14 passages. MW, molecular weight marker, positive control consisted of GFP, YFP, or RFP.

The number of GFP-transformed *E. coli* colonies was noticeably reduced on kanamycin selective media after the third passage and no visible colonies were observed after the 8th passage (Figure 2a). These results were supported by PCR assays that showed amplicons at expected size marker up to the 7th passage (Figure 3). GFP-transformed colonies of *E. cloacae* were visible on the selective media and could be detected by PCR throughout the 14 passages albeit with gradual decrease in colony size and amplicon intensity (Figure 2a and Figure 3). Colonies of GFP-transformed *A. viridans* were detectable on kanamycin selective media up to the 10th passage, but the PCR amplicons were detectable up to 7th passage (Figure 2a and Figure 3). Just like for *E. cloacae*, there was gradual decrease in colony size and amplicon intensity for *A. viridans*. Finally, GFP-labeled *A. hydrophila* consistently produced colonies on selective media and no noticeable change in colony density was detected (Figure 2a). PCR amplicons of GFP-transformed *A. hydrophila* were also consistently detected throughout the 14 passages (Figure 3).

### 3.3. Stability of YFP Plasmids

YFP labeled strains of *E. coli*, *E. cloacae* and *A. hydrophila* consistently produced colonies throughout the 14 passages with no apparent reduction in colony density (Figure 2b). These findings were supported by the detected of PCR amplicons in all 14 passages (Figure 3).

### 3.4. Stability of RFP Plasmids

RFP labeled strains of *E. coli* and *E. cloacae* produced colonies in all 14 passages and no apparent change in colony density was observed (Figure 2). In addition, PCR amplicons at expected size marker were detected in all 14 passages (Figure 3).

## 4. Discussion

Recent studies have shown that bacterial communities residing in mosquito midguts play a critical role in mosquito development, blood meal digestion, strengthening of mosquito immune system, and inhibiting pathogen transmission [3,6,15,26]. Some of these bacterial species can also be genetically modified to express antipathogen effector molecules that reduce vector competence [9,11]. These developments have provided new opportunities for exploiting bacterial function to combat mosquito-borne diseases [27]. However, our understanding of the functional roles of specific members of this microbial complex is still very basic. The overall goal of this study was to label the midgut bacterial isolates from *Oc. triseriatus*, the primary vector of LACV with three fluorescent proteins (GFP, YFP, RFP) to enable us to study their fate and functions in this important vector. We also assessed the stability of these proteins in the labeled bacteria and its influence on bacterial growth. Overall, labeling 4 (*E. coli*, *E. cloacae*, *A. hydrophila* and *A. viridans*) of the 11 bacterial species with at least one of the three fluorescent proteins was readily achieved by electroporation. The four bacterial species are commonly found in guts of various groups of insects and with exception of *A. viridans*, their transformation with GFP has previously been achieved in other systems [28,29]. All four bacterial species were successfully labeled with GFP while three (*E. coli*, *E. cloacae*, and *A. hydrophila*) and two (*E. coli* and *E. cloacae*) bacterial species respectively, were successfully labeled with YFP and RFP. These findings demonstrate that electroporation is a species-specific event and should be used in conjunction with other methods of bacterial transformation to increase the range of bacterial species that can be successfully labeled with any of the three fluorescent proteins. For example, labeling different strains of *E. coli*, *Salmonella*, and *Listeria* with GFP was easily achieved through a combination of electroporation, conjugation, and calcium chloride procedure [20]. Direct imported plasmid DNA generated in electroporation is known to limit the success of transformation because unlike the single stranded intermediates such as those generated by conjugation, it is subject to restriction/modification by the host systems [20]. Thus, combining various methods of bacterial transformations may allow one to overcome the drawbacks associated with each method.

A suitable marker gene for studying the behavior and function of its host bacterial species in mosquito midgut should not be lost during the course of experiment. However, plasmid instability, a tendency of the transformed cells to lose their engineered properties because of changes to or loss of plasmid is one of the most important challenges associated with production of recombinant microorganisms [30]. We subcultured labeled bacterial species for 14 passages to determine the stability of GFP/YFP/RFP markers. YFP- and RFP-labeled bacterial strains were the most stable; they were not only detected by PCR throughout the 14 passages but also exhibited consistent growth during this period. In contrast, GFP-labeled bacterial strains were less stable with GFP-labeled *A. hydrophila* as the only bacterial species that exhibited consistent growth throughout the 14 passages. The two widely-recognized forms of plasmid instability are segregational and structural instability. In segregational instability, a plasmid-free strain outgrows and eventually outcompetes a plasmid-bearing strain resulting in the elimination of the plasmid-bearing strain from the culture [31]. This form of instability is affected by factors such as growth rate of plasmid-bearing and plasmid-free strains, genetic characteristics of host cells, and the stress imposed on the host cells either by the presence of the plasmid or the environment [32]. In contrast, structural instability of plasmids may arise by deletion, insertion, or rearrangement of DNA [33]. Given the slower growth rate of GFP-labeled strains relative to wild type, YFP-, and RFP-labeled strains, it appears that maintenance of GFP-bearing plasmid interfered with normal bacterial growth providing evidence for segregational instability. Further, the failure of GFP-labeled *A. viridans* to grow in the presence of kanamycin suggest the possibility of structural alterations that may have interfered with the function of the kanamycin resistance gene. Collectively, these findings suggest that plasmids bearing YFP and RFP impose less metabolic cost to the host bacteria compared to GFP-bearing plasmids and provide more stable and easily detectable phenotypes for labeling certain bacterial symbionts to study their functions in mosquitoes. Further studies are needed on how to improve the stability of GFP-labeled plasmid and reduce its negative effects on host bacteria to provide three marker genes (GFP, YFP, RFP) that can allow investigations on how combinations of bacterial species interact within the mosquito midgut. These studies should also include evaluation of other GFP variants including the blue and cyan fluorescent proteins as potential markers for a variety of bacterial species isolated from midguts of different mosquito species.

## 5. Conclusions

Overall, our study has successfully transformed four bacterial species whose function in mosquito biology can now be conveniently studied. However, additional studies are needed to improve the success rate of labeling various bacterial isolates from mosquitoes with the three fluorescent proteins. These studies will improve our ability to study bacterial functions in mosquitoes and propel the discovery of bacterial species that can be harnessed for symbiotic control of mosquito-borne diseases.

## Figures and Tables

**Table 1:** Fluorescent protein-specific primers used for the PCR assays. YFP: yellow fluorescent proteins; RFP: red fluorescent proteins.

Target	Primer Name	Sequence	Amplicon
All	UniversalF UniversalR	5-CGA AAC CCG ACA GGA CTA TAA A-3 5-CTA CAT ACC TCG CTC TGC TAA TC-3	328 bp
RFP	RFP1F RFP1R	5-CGA CAT CAA ACT GGA CAT CAC C -3 5-CAC ATG TTC TTT CCT GCG TTA TC-3	344 bp
YFP	YFP1F YFP1R	5-CTG CTT GTC GGC CAT GAT ATA G-3 5-CAT GAA GCA GCA CGA CTT CT-3	241 bp
